# CTLA-4 Engagement Inhibits Th2 but not Th1 Cell Polarisation

**DOI:** 10.1080/10446670310001598519

**Published:** 2003-03

**Authors:** Vanessa Ubaldi, Lucia Gatta, Luigia Pace, Gino Doria, Claudio Pioli

**Affiliations:** 1 Department of Biology University of Tor Vergata Rome I-00133 Italy; 2 Section of Toxicology and Biomedicine ENEA C.R. Casaccia Via Anguillarese 301 Rome I-00060 Italy

## Abstract

CTLA-4 deficient mice show severe lymphoproliferative disorders with T helper sub-population skewed toward the Th2 phenotype. In the present work, we investigated the role of CTLA-4 in T helper cell subset differentiation. Naïve CD4^+^ cells were stimulated with anti-CD3 and anti-CD28 mAbs in the presence of either IL-12 or IL-4 to induce polarisation to Th1 or Th2 cells, respectively. Under these two polarising conditions cells express comparable levels of CTLA-4. CTLA-4 was stimulated by plastic-bound mAb. The frequency of IFN-γ- and IL-4-producing cells were estimated by FACS analysis. In parallel cultures, polarised Th1 and Th2 cells were re-stimulated with anti-CD3 and anti-CD28 mAbs for 48 h and their culture supernatants analysed by ELISA. Results show that CTLA-4 engagement during differentiation inhibits polarisation of naïve CD4^+^ cells to the Th2 but not the Th1 cell subset. At variance, once cells are polarised, CTLA-4 engagement inhibits cytokine production in both effector Th2 and Th1 cells. Altogether these data indicate that CTLA-4 may interfere not only in the signalling involved in acute transcriptional activation of both Th1 and Th2 cells but also in the development of one of the Th cell subsets.


*Clinical & Developmental Immunology*, March 2003, Vol. 10 (1), pp. 13–17

Taylor & Francis healthsciences

### CTLA-4 Engagement Inhibits Th2 but not Th1 Cell Polarisation

VANESSA UBALDI[a], LUCIA GATTA[b], LUIGIA PACE[a], GINO DORIA[a] and CLAUDIO PIOLI[b,*]

[a]*Department of Biology, University of Tor Vergata, I-00133, Rome, Italy;* [b]*Section of Toxicology and Biomedicine, ENEA C.R. Casaccia, Via Anguillarese 301, I-00060, Rome, Italy*

CTLA-4 deficient mice show severe lymphoproliferative disorders with T helper sub-population skewed toward the Th2 phenotype. In the present work, we investigated the role of CTLA-4 in T helper cell subset differentiation. Naïve $CD4^+$ cells were stimulated with anti-CD3 and anti-CD28 mAbs in the presence of either IL-12 or IL-4 to induce polarisation to Th1 or Th2 cells, respectively. Under these two polarising conditions cells express comparable levels of CTLA-4. CTLA-4 was stimulated by plastic-bound mAb. The frequency of IFN-γ- and IL-4-producing cells were estimated by FACS analysis. In parallel cultures, polarised Th1 and Th2 cells were re-stimulated with anti-CD3 and anti-CD28 mAbs for 48 h and their culture supernatants analysed by ELISA. Results show that CTLA-4 engagement during differentiation inhibits polarisation of naïve $CD4^+$ cells to the Th2 but not the Th1 cell subset. At variance, once cells are polarised, CTLA-4 engagement inhibits cytokine production in both effector Th2 and Th1 cells. Altogether these data indicate that CTLA-4 may interfere not only in the signalling involved in acute transcriptional activation of both Th1 and Th2 cells but also in the development of one of the Th cell subsets.

*Keywords*: Costimulation; CTLA-4; Cytokines; IL-4; T helper differentiation

### INTRODUCTION

TCR ensures antigen specificity and MHC restriction of T cell activation. Additional signals delivered by co-stimulatory molecules and cytokines sustain and integrate TCR signalling resulting in optimal cell proliferation and differentiation. Upon activation, naïve CD4 cells differentiate in subsets displaying different cytokine patterns. Th1 cells produce IL-2 and IFN-γ, and promote cell-mediated immune responses, whereas Th2 cells secrete IL-4, IL-5, IL-6 and IL-13, and sustain humoral responses. Micro-environment/milieu at priming conditions naïve $CD4^+$ differentiation. IL-12 produced by dendritic cells induces Th1 differentiation, whereas IL-4 produced by other T cells promotes differentiation to Th2 cells. In addition to cytokines, several factors such as intensity of TCR signal, costimulation, type of APC and genetic background have been shown to affect Th cell differentiation (Abbas *et al.*, 1996; Constant and Bottomly, 1997; O'Garra, 1998).

CD28, the main co-stimulatory receptor, plays an important role in T cell activation up-regulating cytokine production, CD25 expression and cell proliferation. CD28 engagement by CD80 (B7.1) and CD86 (B7.2) expressed on APC, promotes Th2 subset differentiation *in vitro* (Webb and Feldmann, 1995; Rulifson *et al.*, 1997) and *in vivo* (King *et al.*, 1996). A second receptor for B7 molecules, CTLA-4 (CD152), is up-regulated on activated T cells with maximum of expression at 48 h. It exerts a negative role in T cell activation inhibiting factors required for cytokine production and cell proliferation (Krummel and Allison, 1996; Walunas *et al.*, 1996; Pioli *et al.*, 1999). The critical role played by this receptor is evident in CTLA-4 deficient mice which develop dramatic lymphoproliferative disorders and die by 1 month of age (Tivol *et al.*, 1995; Waterhouse *et al.*, 1995). In these mice, all immunoglobulin isotypes show higher basal serum levels as compared to wild-type mice, ranging from 10-fold for IgM, $IgG_{2a}$, $IgG_{2b}$ and $IgG_3$, to 100-fold for $IgG_1$ and IgA, to several thousand fold for IgE (Waterhouse *et al.*, 1995). CD4 cells from CTLA-4 deficient mice display higher cytokine production as compared to the wild type counterpart, IL-4 being much more increased than IFN-γ production (Khattri *et al.*, 1999). Moreover, lack of CTLA-4 during antigen-dependent activation in transgenic CD4 cells results in differentiation to Th2 cells (Oosterwegel *et al.*, 1999). These data all together prompted us to investigate the role of CTLA-4 during T helper cell polarisation.

*Corresponding author. Tel.: +39-06-3048-3398. Fax: +39-06-3048-3644. E-mail: pioli@casaccia.enea.it

ISSN 1740-2522 print/ISSN 1740-2530 online © 2003 Taylor & Francis Ltd
DOI: 10.1080/10446670310001598519

### MATERIALS AND METHODS

### Cell Purification and Cultures

Splenic naïve $CD4^+$ T cells from C57Bl/6 mice were purified by immunomagnetic cell sorting according to the manufacturer's instructions (Miltenyi, catalogue numbers 130-058-701 and 130-049-701). Collected cells were found to be almost exclusively (>95%) $CD4^+CD45RB^{hi}$ by flow cytometry analysis. To induce Th cell polarisation, naïve $CD4^+$ cells were cultured in plates pre-coated with anti-CD3ε mAb (clone 145-2C11; 10 μg/ml). Anti-CD28 mAb (clone 37.51) was used in soluble form (1 μg/ml). In Th1 polarising cultures, IL-12 (10 ng/ml) and an anti-IL-4 blocking mAb were added. In Th2 polarising cultures, IL-4 (5 ng/ml) and an anti-IFN-γ blocking mAb were added. Anti-CTLA-4 mAb (clone UC10-4F10-11) or hamster IgG (isotype control) was bound to plates at the concentration of 10 μg/ml. Polarised cells were re-stimulated with PMA and ionomycin for 5 h, in the presence of brefeldin A, or for 48 h to analyse the frequency of cytokine-producing cells and cytokine release in supernatants, respectively. Polarised cells were also re-stimulated with bound anti-CD3 and soluble anti-CD28 mAb for 48 h. All the antibodies employed in culture were sodium azide- and endotoxin-free as certified by the producer (Pharmingen).

### Cytokine Titration

Cytokines were titrated in culture supernatants by sandwich ELISA as already described (Pioli *et al.*, 1998). The reference straight line obtained by plotting the absorbance versus the standard cytokine concentrations was used to calculate cytokine concentrations in the supernatants.

### Flow Cytometry Analysis

Cells were pre-incubated with Fc Block (anti-CD16/32, clone 2.4G2) to prevent cytophilic binding of labelled Abs. Cytokine-producing cells were revealed by double-staining cells with PE-conjugated anti-IL-4 mAb (clone BVD4-1D11) and FITC-conjugated anti-IFN-γ mAb (clone XMG1.2). Intracellular CTLA-4 was revealed with a PE-conjugated anti-CTLA-4 mAb (clone UC10-4F10-11). Before staining cells were permeabilised with saponin (5%) and fixed with paraformaldehyde (2%). PE/FITC-conjugated isotype-matched Abs were used as controls. The optimal concentrations of all the Abs were assessed in preliminary experiments. Samples of viable $20 \times 10^4$ cells were analysed, and fluorescence signals collected in log mode using a FACScalibur (Becton Dickinson).

### RT-PCR

Total RNA extracted by TRIzol Reagent (Life Technologies) was used as template for cDNA synthesis performed by Perkin-Elmer GeneAmp RNA PCR kit. After an initial 5-min denaturation at 94°C, the cDNA was amplified for 35 cycles; each cycle was programmed for denaturation at 94°C for 45 s, annealing at 48°C for 60 s, and elongation at 72°C for 90 s. The following oligonucleotides were used as primers: b-actin forward, 5′-CTGAAGTACCCATTGAACATGGC; b-actin reverse, 5′-CAGAGCAGTAATCTCCTTCTGCAT; IL-4 mRNA forward, 5′-TATTGATGGGTCTCAACCCC; IL-4 mRNA reverse, 5′-TCCATTTGCATGATGCTCTT.

### RESULTS

### Cytokine Production and CTLA-4 Expression during Th Cell Polarisation

Purified naïve $CD4^+$ cells were stimulated with anti-CD3, anti-CD28 in the presence of IL-12 and anti-IL-4 blocking mAb and IL-12 to induce differentiation toward Th1 cells (hereafter Th1 condition). Differentiation to Th2 cells was induced by anti-CD3, anti-CD28, IL-4 and anti-IFN-γ blocking mAb (hereafter Th2 condition). To verify that naïve $CD4^+$ cells differentiating to Th1 and Th2 cells were actually producing polarised cytokines we analysed culture supernatants by ELISA and/or mRNA expression by RT-PCR. Under Th1 condition $CD4^+$ cells produced large amounts of IFN-γ whereas IL-5 and IL-10 were barely detectable (Fig. 1, panel A). Under Th2 condition, IL-4 produced by $CD4^+$ cells cannot be distinguished from the IL-4 we added in culture and thus, we analysed IL-4 mRNA expression by RT-PCR. Results show that during Th2 polarisation, $CD4^+$ cells express high levels of IL-4 mRNA (panel B), which increases with the stimulation time, and produce IL-5 and IL-10 but not IFN-γ (panel A).

In $CD4^+$ cell bulk cultures CTLA-4 expression increases with stimulation time reaching a maximum after 48–72 h. Under polarising conditions, naïve $CD4^+$ cells might be induced by IL-12 or IL-4 to express different levels of CTLA-4. To verify this hypothesis naïve $CD4^+$ cells were stimulated under Th1 or Th2 conditions and analysed for CTLA-4 expression by intracellular and surface staining at different stimulation times. As shown in Fig. 1, panels C and D, Th1 and Th2 polarising conditions induce comparable levels of intracellular CTLA-4 expression. Also the kinetics is similar in both groups, the expression being higher at 3–5 days and lower after 1 week of stimulation. Unstimulated naïve $CD4^+$ cells do not express CTLA-4. Surface staining was barely detectable under both Th1 and Th2 polarising conditions (not shown).

### CTLA-4 Engagement Inhibits Th2 but not Th1 Cell Polarisation

Purified naïve $CD4^+$ cells were stimulated under Th1 or Th2 polarising conditions in the presence of either plastic-bound

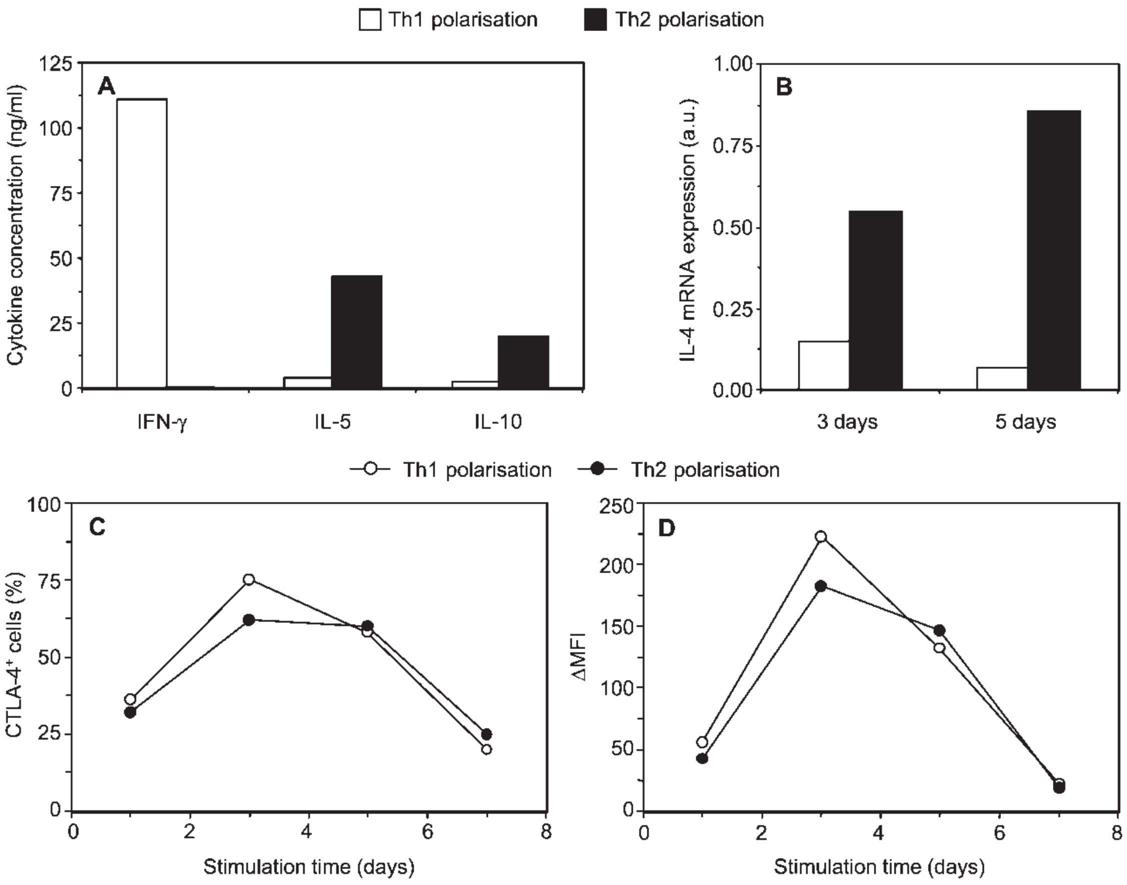

FIGURE 1 Cytokine production and CTLA-4 expression during cell polarisation. Naive $CD4^+$ cells were stimulated under Th1 or Th2 polarising conditions for 3, 5 or 7 days. (A) After 1 week, stimulation cytokine concentration of culture supernatants was evaluated by ELISA. (B) After 3 and 5 days stimulation IL-4 mRNA expression was evaluated by RT-PCR. Amplified products were fractionated in a 2% ethidium bromide-stained agarose gel. Optical density of IL-4 mRNA bands were normalised to optical density of corresponding β-actin samples and values expressed as arbitrary units. (C) After 3, 5 and 7 days stimulation cells were permeabilised, fixed and intracellularly stained with PE-conjugated anti-CTLA-4 mAb. Values represent percentage of positive cells. Not specific staining was evaluated with a isotype-matched PE-conjugated control mAb. (D) CTLA-4 mean fluorescence intensity. Each value represents the difference between the mean fluorescence intensity of the sample stained with the PE-conjugated anti-CTLA-4 mAb and the relevant sample stained with PE-conjugated control mAb.

anti-CTLA-4 mAb or hamster IgG (isotype control). After 1 week, polarisation cells were collected and re-stimulated with PMA and ionomycin for 5 h in the presence of brefeldin A to induce massive cytokine production and retention in intracellular compartments. Cells were then doubled-stained with FITC-conjugated anti-IFN-γ and PE-conjugated anti-IL-4 or isotype control mAbs (Table I).

TABLE I CTLA-4 engagement inhibits polarisation to Th2 cell subset

| Polarising stimuli | Cytokine producing cells[(a)](%) | | | Cytokine production[(b)](ng/ml) | | |
|---|---|---|---|---|---|---|
| | $IL\text{-}4^+$ | $IFN\text{-}\gamma^+$ | $IL\text{-}4^+$ $IFN\text{-}\gamma^+$ | IL-4 | IL-5 | IFN-γ |
| Th1 + iso ctrl | 1.1 | 28.3 | 0.8 | 2.1 | 0.5 | 29.9 |
| Th1 + αCTLA-4 | 0.9 | 30.0 | 0.8 | 1.8 | 0.7 | 32.6 |
| Th2 + iso ctrl | 29.1 | 0.8 | 0.7 | 16.3 | 4.8 | 2.4 |
| Th2 + αCTLA-4 | 15.2 | 1.3 | 1.0 | 8.7 | 3.2 | 2.7 |

Th1 and Th2 polarised cells were re-stimulated with PMA and ionomycin either [(a)] for 5 h in the presence of brefeldin A, double-stained with PE-conjugated anti-IL-4 and FITC-conjugated anti-IFN-γ mAb and analysed by flow cytometry or [(b)] for 48 h and their culture supernatants analysed by ELISA. Iso ctrl: isotype control mAb; αCTLA-4: anti-CTLA mAb.

Flow cytometry analyses show that under Th1-polarising conditions 28% of the cell population produce IFN-γ whereas very few cells (1.1%) produce IL-4 and apparently no one produce both cytokines. CTLA-4 engagement during Th1 polarisation does not affect the frequency of cytokine-producing cells. In parallel cultures, polarised cells were re-stimulated with PMA and ionomycin for 48 h. ELISA reveals that CTLA-4 engagement during Th1 polarisation does not affect IFN-γ production in re-stimulated cells. These data all together indicate that CTLA-4 does not affect differentiation of naïve $CD4^+$ cells to Th1.

Conversely, when naïve $CD4^+$ cells are driven to differentiate toward Th2 cells, CTLA-4 engagement inhibits polarisation. CTLA-4 engagement, indeed, reduces the frequency of IL-4 producing cells from 29 to 15% but without increasing IFN-γ-producing cells (Table I). Upon re-stimulation for 48 h, cells polarised in the presence of anti-CTLA-4 reveal a lower production of IL-4 and IL-5 as compared to control cells. The percentage of inhibition for IL-4 and IL-5 is 46 and 32%, respectively. Thus, CTLA-4 inhibits Th2 but not Th1 cell polarisation.

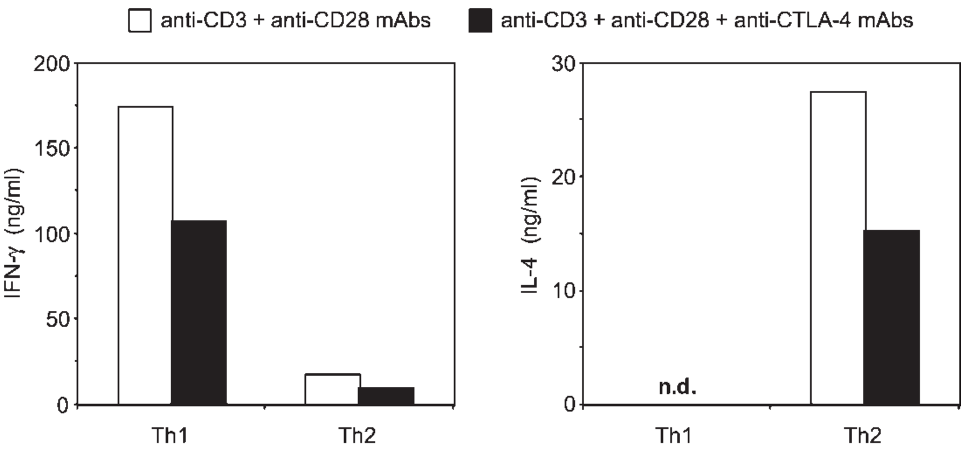

FIGURE 2 Polarised Th1 and Th2 cells were re-stimulated with anti-CD3 and anti-CD28 mAb in the presence of either isotype control or anti-CTLA-4 mAb. After 48 h culture supernatants were analysed by ELISA (n.d., not detectable).

### CTLA-4 Inhibits the Production of Effector Cytokines in both Th1 and Th2 Cells

Polarised Th1 and Th2 cells were re-stimulated with anti-CD3 and anti-CD28 mAb in the presence of either plastic-bound anti-CTLA-4 mAb or hamster IgG (isotype control). After 48 h, culture supernatants were analysed by ELISA. Figure 2 shows that CTLA-4 engagement inhibits cytokine production according to the Th specificity. IL-4 production was, indeed, inhibited in Th2 cells as well as IFN-γ production in Th1 cells. Regardless of CTLA-4 engagement, culture supernatants from re-stimulated Th1 and Th2 cells were negative for IL-4 and IFN-γ, respectively. These data suggest that CTLA-4 is functional in both Th1 and Th2 cells and that it can negatively regulate effector cytokine production in both polarised Th1 and Th2 cells.

### DISCUSSION

CTLA-4 has been widely described to play a negative role in T cell activation (Alegre *et al.*, 2001). T cell CTLA-4 engagement, indeed, inhibits cytokine production, CD25 expression, and cell cycle progression (Walunas *et al.*, 1996; Brunner *et al.*, 1999). Coherently, CTLA-4 blockade enhances T cell responses to Ags (Kearney *et al.*, 1995) and tumours (Leach *et al.*, 1996) and exacerbates auto-immune diseases (Karandikar *et al.*, 1996; Perrin *et al.*, 1996). The timing for the inhibitory effect of CTLA-4 engagement depends on the kinetics of CTLA-4 expression. This receptor, indeed, reaches a maximum of expression after 48 h stimulation, indicating a crucial role in shutting down T cell activities, after the effector function has been turned on. In effector $CD4^+$ cells CTLA-4 inhibits both IFN-γ and IL-4 production, as previously described (Alegre *et al.*, 2001) and reported in the present paper (Fig. 2). On the other hand, the Th2 skewed phenotype of CTLA-4 deficient mice (Khattri *et al.*, 1999) raises the question as to whether CTLA-4 might play a role also in Th cell subset differentiation. In the present paper, we show that CTLA-4 engagement during naïve $CD4^+$ cell polarisation blocks differentiation to Th2 but not to Th1 cell subset. Upon re-stimulation, indeed, CD4 cells cultured for 1 week under Th2 polarising conditions in the presence of anti-CTLA-4 mAb show reduced frequency of IL-4-producing cells and IL-4 and IL-5 production as compared to control group (Table I). At variance, CTLA-4 engagement during polarisation does not affect the frequency of IFN-γ-producing cells. This contrast could be due to a different effect of CTLA-4 signals on transduction pathways leading to Th1/Th2 differentiation. CTLA-4 has been described to interfere with signals delivered by the TCR/CD3 complex inhibiting LAT phosphorylation (Lee *et al.*, 1998). Inhibition of LAT activation could explain inhibitory effects on downstream factors required for "acute" gene transcription as it occurs in already polarised (effector) $CD4^+$ cells. Cytokine mRNA expression induced by TCR/CD28 costimulation in polarised cells, indeed, occurs according to the cell subset specificity also in the absence of polarising cytokines (IL-12 or IL-4) (Table I and Fig. 2). During polarisation, in the presence of IL-12 or IL-4, other signalling pathways are also activated. Differentiation is reached by chromatin remodelling driven by polarising cytokines. Under Th2 polarising conditions, this process leads to a better accessibility of the IL-4/IL-5/IL-13 locus for transcription factors and to a reduced accessibility of the IFN-γ promoter. It is possible that CTLA-4 interferes with the activation of factors required for this process. Alternatively, CTLA-4 by interfering with signals generated by CD3/CD28 co-stimulation might block Th2 cell differentiation sustained by CD28 stimulation. In a physiological process, competition between CTLA-4 and CD28 for binding to B7 ligands could also play role, especially when B7 expression is limiting. We are currently investigating these mechanisms.

In conclusion, our work shows that CTLA-4 plays a dual role. It controls the production of effector cytokines in both Th1 and Th2 cells whereas it affects only naïve CD4 cell differentiation to Th2.

### *References*

Abbas, A.K., Murphy, K.M. and Sher, A. (1996) "Functional diversity of helper T lymphocyte", *Nature* **383**, 787–793.

Alegre, M.L., Frauwirth, K.A. and Thompson, C.B. (2001) "T-cell regulation by CD28 and CTLA-4", *Nat. Rev. Immunol.* **1**, 220–228.

Brunner, M.C., Chambers, C.A., Chan, F.K., Hanke, J., Winoto, A. and Allison, J.P. (1999) "CTLA-4-mediated inhibition of early events of T cell proliferation", *J. Immunol.* **162**, 5813–5820.

Constant, S.L. and Bottomly, K. (1997) "Induction of Th1 and Th2 CD4 T cell responses: the alternative approaches", *Annu. Rev. Immunol.* **15**, 297–322.

Karandikar, N.J., Vanderlugt, C.L., Walunas, T.L., Miller, S.D. and Bluestone, J.A. (1996) "CTLA-4: a negative regulator of autoimmune disease", *J. Exp. Med.* **184**, 783–789.

Kearney, E.R., Walunas, T.L., Karr, R.W., Morton, P.A., Loh, D.Y., Bluestone, J.A. and Jenkins, M.K. (1995) "Antigen-dependent clonal expansion of a trace population of antigen-specific CD4 T cells *in vivo* is dependent on CD28 costimulation and inhibited by CTLA-4", *J. Immunol.* **155**, 1032–1036.

Khattri, R., Auger, J.A., Griffin, M.D., Sharpe, A.H. and Bluestone, J.A. (1999) "Lymphoproliferative disorder in CTLA-4 knockout mice is characterized by CD28-regulated activation of Th2 responses", *J. Immunol.* **162**, 5784–5791.

King, C.L., Xianli, J., June, C.H., Abe, R. and Lee, K.P. (1996) "CD28-deficient mice generate an impaired Th2 response to *Schistosoma mansoni* infection", *Eur. J. Immunol.* **26**, 2448–2455.

Krummel, M.F. and Allison, J.P. (1996) "CTLA-4 engagement inhibits IL-2 accumulation and cell cycle progression upon activation of resting T cells", *J. Exp. Med.* **183**, 2533–2540.

Leach, D.R., Krummel, M.F. and Allison, J.P. (1996) "Enhancement of antitumor immunity by CTLA-4 blockade", *Science* **271**, 1734–1736.

Lee, K.M., Chuang, E., Griffin, M., Khattri, R., Hong, D.K., Zhang, W., Straus, D., Samelson, L.E., Thompson, C.B. and Bluestone, J.A. (1998) "Molecular basis of T cell inactivation by CTLA-4", *Science* **282**, 2263–2268.

O'Garra, A. (1998) "Cytokines induce the development of functionally heterogeneous T helper cell subsets", *Immunity* **8**, 275–283.

Oosterwegel, M.A., Mandelbrot, D.A., Boyd, S.D., Lorsbach, R.B., Jarrett, D.Y., Abbas, A.K. and Sharpe, A.H. (1999) "The role of CTLA-4 in regulating Th2 differentiation", *J. Immunol.* **163**, 2634–2639.

Perrin, P.J., Maldonado, J.H., Davis, A.T., June, C.H. and Racke, M.K. (1996) "CTLA-4 blockade enhances clinical disease and cytokine production during experimental allergic encephalomyelitis", *J. Immunol.* **157**, 1333–1336.

Pioli, C., Pucci, S., Barile, S., Frasca, D. and Doria, G. (1998) "Role of mRNA stability in the different patterns of cytokine production by CD4 cells from young and old mice", *Immunology* **94**, 380–387.

Pioli, C., Gatta, L., Frasca, D. and Doria, G. (1999) "Cytotoxic T lymphocyte antigen 4 (CTLA-4) inhibits CD28-induced IκBα degradation and RelA activation", *Eur. J. Immunol.* **29**, 856–863.

Rulifson, I.C., Sperling, A.I., Fields, P.E., Fitch, F.W. and Bluestone, J.A. (1997) "CD28 costimulation promotes the production of Th2 cytokines", *J. Immunol.* **158**, 658–665.

Tivol, E.A., Borriello, F., Schweitzer, A.N., Lynch, W.P., Bluestone, J.A. and Sharpe, A.H. (1995) "Loss of CTLA-4 leads to massive lymphoproliferation and fatal multiorgan tissue destruction, revealing a critical negative regulatory role of CTLA-4", *Immunity* **3**, 541–547.

Walunas, T.L., Bakker, C.Y. and Bluestone, J.A. (1996) "CTLA-4 ligation blocks CD28-dependent T cell activation", *J. Exp. Med.* **183**, 2541–2550.

Waterhouse, P., Penninger, J.M., Timms, E., Wakeham, A., Shahinian, A., Lee, K.P., Thompson, C.B., Griesser, H. and Mak, T.W. (1995) "Lymphoproliferative disorders with early lethality in mice deficient in Ctla-4", *Science* **270**, 985–988.

Webb, L.M. and Feldmann, M. (1995) "Critical role of CD28/B7 costimulation in the development of human Th2 cytokine-producing cells", *Blood* **86**, 3479–3586.

